# Bridging the clinical trial gap in Saudi Arabia: a multi-stakeholder conference-based consensus on strategic initiatives for global competitiveness and patient inclusion

**DOI:** 10.1007/s44446-026-00099-7

**Published:** 2026-07-21

**Authors:** Majed Al Jeraisy, Eva Turgonyi, Vladimir Misik, Ahmed Alaskar

**Affiliations:** 1 College of Pharmacy, King Saud Bin Abdulaziz University for Health Sciences, Ministry of National Guard, Health Affairs, Riyadh, Saudi Arabia; 2 King Abdullah International Medical Research Center (KAIMRC), Ministry of National Guard-Health Affairs (MNGHA), Riyadh, Saudi Arabia; 3 Present Address: Saudi National Health Institute, Riyadh, Saudi Arabia; 4 GCC (Gulf Countries Cluster) & Pakistan, AstraZeneca, Dubai, United Arab Emirates; 5 LongTaal Clinical Research Institute, Bratislava, Slovakia; 6 DUNA.Logic – Clinical Research Informatics, Hainburg/Donau, Austria; 7 Adult Hematology & HSCT, King Saud Bin Abdulaziz University for Health Sciences, Ministry of National Guard, Health Affairs, Riyadh, Saudi Arabia

**Keywords:** Clinical research inequity, Consensus statement, Clinical trials, Saudi Arabia, Vision 2030, R&D investment

## Abstract

**Background:**

The clinical trial ecosystem of Saudi Arabia has advanced significantly, with innovative industry-sponsored clinical trials (iCTs) growing by 27% from 2018 to 2023, reaching 51.4% in the first half of 2025. However, the Kingdom remains underrepresented in global research. This study aimed to quantify this gap and develop strategies to enhance Saudi Arabia's competitiveness.

**Methods:**

A mixed-methods research design was employed. Phase 1 involved a quantitative landscape analysis of historical and current iCT data (2016–2023) using the LongTaal Clinical Trial Informatics platform. Phase 2 involved a qualitative assessment of expert input derived from two national clinical trial conferences (2022 and 2024), in which expert panel discussions and documented outputs were used to contextualize findings and inform strategic recommendations.

**Results:**

In 2023, Saudi Arabia’s iCT market share was < 0.06%, significantly lower than its ~ 0.9% share of global pharmaceutical consumption. This 16-fold disparity highlights a critical lack of patient representation. Economic modelling indicates that achieving parity could increase annual research and development (R&D) investment from ~ USD 46 million to over USD 740 million. Insights derived from conference-based expert discussions highlighted regulatory timelines, infrastructure fragmentation, and insufficient incentives as primary barriers.

**Conclusion:**

To bridge this gap, the authors propose operationalizing a centralised national body to serve as a "one-stop shop" for global sponsors. Strategic priorities include harmonizing regulatory timelines to 6 months, establishing distributed Centres of Excellence, and implementing direct R&D financial incentives. These measures are essential to ensuring Saudi patients are adequately represented in the development of novel pharmaceuticals.

**Supplementary Information:**

The online version contains supplementary material available at https://doi.org/10.1007/s44446-026-00099-7.

## Background

**Fig. 1:**
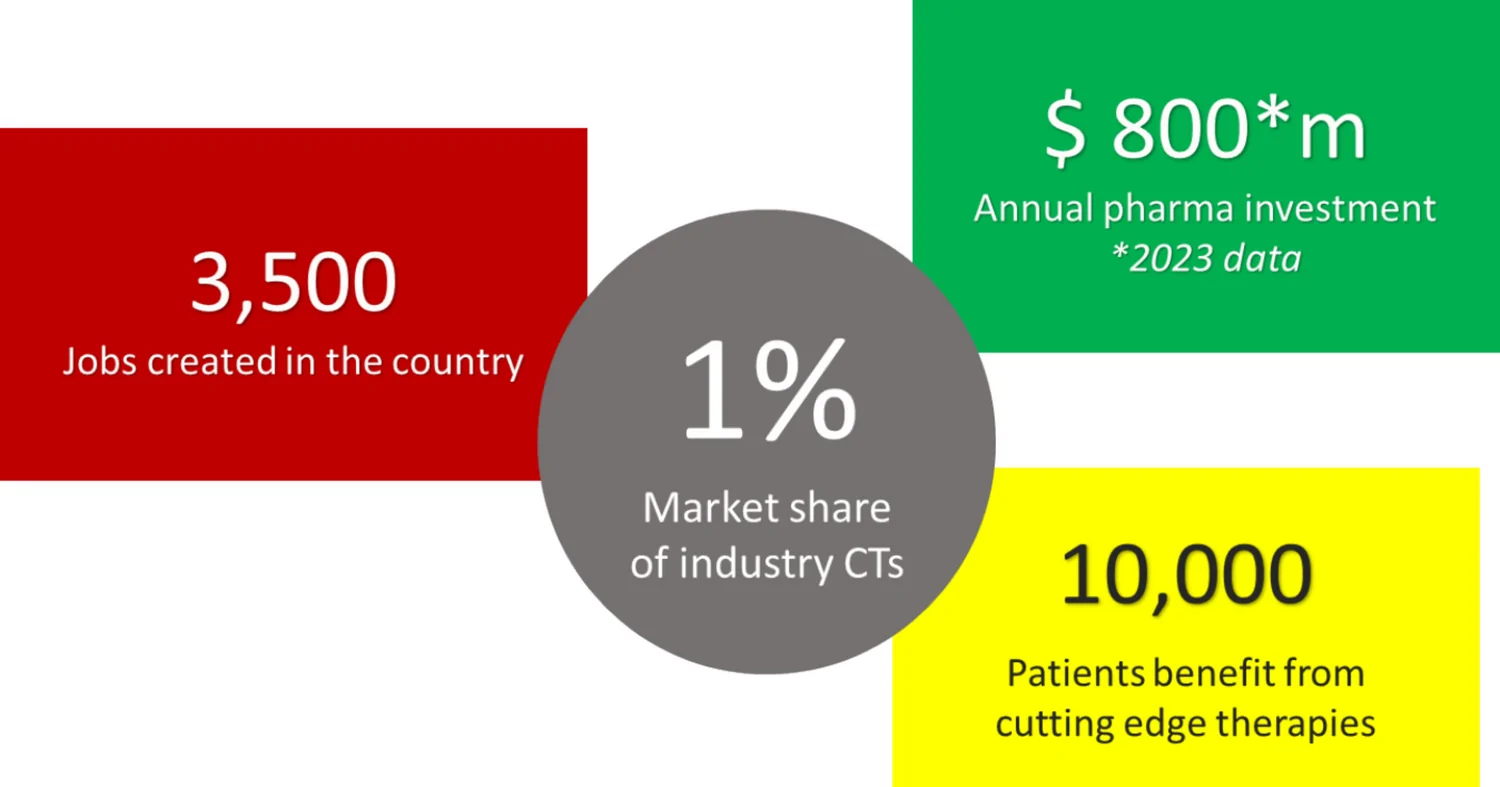
Socioeconomic value of 1% global market share of iCTs (2023 data)

Innovative industry-sponsored clinical trials (iCTs) constitute a fundamental pillar of contemporary biomedical research, offering substantial advantages to patients, healthcare professionals, and healthcare systems (Fig. 1). From the patient's perspective, iCTs provide early access to investigational therapies that may otherwise remain unavailable for several years, particularly in the context of complex or underserved disease areas. Participation in iCTs also ensures clinical oversight, with frequent monitoring and comprehensive follow-up protocols, potentially enhancing patient safety and outcomes (Misik et al. 2014). Additionally, clinicians gain first-hand experience with novel interventions and improve their research capabilities, while healthcare institutions benefit from opportunities to develop centres of excellence. Also, it has been shown that institutions that regularly participate in clinical trials achieve improved patient outcomes in routine clinical practice (Boaz et al. 2015; Downing et al. 2017; Jonker et al. 2020). The economy also profits through job creation, direct research and development (R&D) investments, and taxation. For example, Poland saw USD 1.3 billion generated by clinical trials in 2019 (Misik et al. 2021), while over the period 2016–2019, clinical trials added GBP 8 billion to the United Kingdom (UK) economy and created 47,467 full-time equivalent (FTE) jobs (EVERSANA 2024).

Over the past three decades, pharmaceutical companies have steadily shifted their clinical trial operations away from North America and Western Europe, largely prompted by the adoption of ICH Good Clinical Practice (ICH GCP) in 1997. Notably, North America and Western Europe's share of global trial sites dropped from 94% in 1995 to below 80% by 2005 (Glickman et al. 2009), while pivotal-trial recruitment declined from 80% in 2005 to 66% by 2008 (European Medicines Agency 2010). Between 2008 and 2024, they lost a further 13% of their trial sites to other parts of the world (LongTaal, n.d.). These moves reflect industry-wide efforts to expand the global clinical trial footprint, reduce costs, and accelerate drug development. This shift provides opportunities for emerging markets to become R&D hubs.

Despite this shift, sponsors and contract research organisations (CROs) commonly encounter difficulties in completing clinical trials on schedule and within budget, primarily due to recruitment shortfalls. Around 55% of studies terminate prematurely due to insufficient enrolment, and globally, more than 80% fail to meet recruitment targets or timelines (Desai 2020; Wandile 2023). Contributing factors include restrictive eligibility criteria, logistical barriers, and high patient financial burdens (Czobor and Skolnick 2011; Chaudhry and Trailokya 2023). Consequently, sponsors are generally reluctant to expand into new settings; they favour established sites offering a proven recruitment track record and operational efficiencies.

However, this cautious approach conflicts with current regulations to enhance racial and ethnic diversity in clinical trials. The United States (US) Food and Drug Administration (FDA) has highlighted the persistent underrepresentation of specific demographic groups whose higher disease burdens are not adequately captured in existing datasets, and sponsors are being urged to adopt strong strategies to ensure adequate recruitment diversity (U.S. Food and Drug Administration, n.d.). Other global regulators, including the European Medicines Agency (EMA), the UK's Medicines and Healthcare Products Regulatory Agency (MHRA), and Health Canada, have emphasised the importance of ensuring racial and ethnic diversity in novel drug development and marketing approvals. Nonetheless, populations in regions like the Middle East remain underrepresented in pivotal drug development (Nasser et al. 2023). Previous work has documented the substantial gap in trial enrolment among Arabic-speaking populations (Mišík et al. 2017a; Misik and Bolecek 2023). As global demand for more inclusive and representative data rises, attracting iCTs becomes a strategic priority. Diverse clinical data are essential to accurately evaluate the effectiveness and safety of new therapies across different ethnic populations with distinct genetic backgrounds, enabling the development of truly effective and safe medicines for all patient groups. Consequently, finding effective mechanisms to lower operational barriers, streamline regulatory pathways, and foster clinical trial expertise is essential for bridging these diversity gaps.

### Current clinical trials landscape in Saudi Arabia

In recent years, Saudi Arabia has established an ambitious transformation agenda under the Vision 2030 framework (Kingdom of Saudi Arabia, n.d.). This strategy aims to diversify the nation's economy by reducing its reliance on oil revenues and fostering growth in a range of non-oil sectors, including healthcare, technology, renewable energy, and tourism. The National Biotechnology Strategy (2024) has four key pillars: innovative vaccine technologies, biomanufacturing and localisation, genomics, and food self-sufficiency and agricultural productivity. The objective of the Economic Participation Policy is to support the development of new capabilities and attract new investments, with one of its highlighted categories being R&D, which includes financial incentives to sponsor companies.

The pharmaceutical sector is among the primary beneficiaries of this drive. Saudi Arabia's pharmaceutical market, estimated at USD 12.37 billion in 2025, is expected to reach USD 18.12 billion by 2030 with an estimated compound annual growth rate (CAGR) of around 7.93% (MarkNtel Advisors 2025). From 2019 to 2023, the Kingdom's pharmaceutical sector grew at an average annual rate of 6%, from USD 8 billion to USD 10 billion.

Several recent developments suggest that Saudi Arabia is poised to enhance its standing in iCTs (Alsaywid et al. 2024). The Saudi National Institutes of Health (SNIH) has taken on an expanded role as a national clinical research facilitator. Simultaneously, the newly established Saudi Clinical Trials Enterprise (SCTE) focuses on training and upskilling the clinical trials workforce, comprising investigators, study nurses, coordinators, and pharmacists, and promoting best practices at trial sites (King Abdullah International Medical Research Centre (KAIMRC) 2024). The recent launch of the SNIH's Clinical Trials Acceleration initiative indicates that government entities and key institutions recognise the need to expand the clinical trial ecosystem. Guided by the SNIH in collaboration with the Ministry of Investment (MISA), the Ministry of Health (MoH), research centres, healthcare institutions, and the Saudi FDA (SFDA), this initiative is designed to streamline the clinical trial process, strengthen research infrastructure, and position Saudi Arabia as a premier hub for global R&D trials. The initiative aims to foster a research-friendly environment that enhances innovation and patient access to cutting-edge treatments by attracting multinational pharmaceutical companies, biotechnology firms, and academic partnerships.

In the short term, the initiative focuses on transforming active research sites into Centres of Clinical Research Excellence that meet international standards, thereby expanding the national clinical trials footprint by 2027 and enhancing the Kingdom's capacity to conduct high-quality trials. Equipping these centres with Clinical Trial Management Systems (CTMS), trained personnel (including investigators, study coordinators, and specialised clinical research nurses), and advanced digital tools, such as Electronic Medical Records (EMR) data-mining technology and artificial intelligence (AI)-driven analytics, is intended to facilitate patient recruitment and optimise study designs. In the longer term, this strategy envisions a state-of-the-art clinical trial infrastructure that offers a favourable regulatory environment, streamlined Clinical Trial Agreement (CTA) submission, competitive trial start-up times (within 6 months) and financial incentives. The initiative could position Saudi Arabia as a regional leader in clinical research, particularly in oncology, rare diseases, and gene therapy.

However, despite its robust economy, substantial investments in healthcare, and the launch of the Clinical Trials Acceleration initiative, a significant "implementation gap" remains. Saudi Arabia continues to be disproportionately underrepresented in global iCTs relative to its pharmaceutical consumption and economic standing. This persistence suggests that structural and operational barriers remain that cannot be addressed by policy announcements alone, but require coordinated, multi-stakeholder intervention.

To address these persistent gaps, the SCTE convened the 1 st and 2nd Regional Clinical Trial Conferences in Riyadh (December 2022 and March 2024). These forums brought together policymakers, regulators, industry leaders, and clinical investigators to move beyond landscape analysis and agree on a unified course of action. Despite the great efforts and initiatives done by the SCTE to unite the stakeholders to improve the status of the country’s reputation, the national impact on the clinical trials ecosystem had still not existed.

This manuscript presents findings derived from this process, integrating a quantitative benchmarking analysis (covering the 2016–2023 period) with the strategic insights informed by conference-based expert discussions. The objective is not merely to report on the current status but also to provide an expert-endorsed roadmap to bridge the diversity gap and ensure that Saudi patients are adequately represented in the global development of novel biopharmaceuticals.

## Methods

### Study design

A mixed-methods research design was adopted, integrating quantitative analysis of the global and national clinical trial landscape with qualitative assessment of expert input derived from national conference proceedings involving key national stakeholders. The methodological framework comprised two distinct phases. Phase 1 consisted of a quantitative landscape analysis to establish baseline evidence regarding Saudi Arabia's market share and patient inclusion in iCTs. Phase 2 involved a retrospective qualitative review of expert panel discussions and documented outputs (meeting minutes and summary reports) from two national clinical trial conferences. This phase aimed to contextualise the quantitative findings, identify challenges and priorities, and inform the development of strategic recommendations.

### Phase 1: Quantitative landscape analysis (Evidence Generation)

To provide an evidence base for the expert discussions, historical and current iCT data covering the period from 2016 to 2023 were analysed using the LongTaal Clinical Trial Informatics platform (LongTaal, n.d.). This platform was selected for its ability to integrate and standardize data from ClinicalTrials.gov and the EU Clinical Trials Register.

#### Market share calculation

This study calculated market share based on active clinical trial sites to reflect ongoing research activity, rather than relying on new trial registrations. Market share was defined as the percentage of active iCT sites in Saudi Arabia relative to the global total.

#### Patient accessibility and consumption

Patient accessibility was assessed by calculating the number of iCT sites per one million population, indexed against US levels. Furthermore, a Participation to Consumption Ratio (PCR) was computed to quantify Saudi patients' representation in drug development relative to their consumption of innovative medicines. This was achieved by comparing the country's share of global iCT sites against its share of global pharmaceutical sales.

#### Socioeconomic impact

Economic modelling was applied to estimate R&D investment, job creation, and patient access, utilizing global industry benchmarks (e.g., average spend per trial site) (Taylor et al. 2020; Simoens and Huys 2021; Andersen et al. 2023; Chandra et al. 2024).

### Phase 2: Qualitative assessment of conference-derived expert opinion

Phase 2 involved a retrospective assessment of expert input derived from two national clinical trial conferences: the first Regional Clinical Trial Conference (December 2022) and the second International Clinical Trial Conference (March 2024), both hosted by the King Abdullah International Medical Research Centre (KAIMRC) in Riyadh, Saudi Arabia. Expert input was obtained through review of documented conference materials, including meeting minutes, panel discussions, and summary statements generated during these events.

Conference attendees represented a broad range of stakeholders across the Saudi clinical trial ecosystem, including regulatory authorities (SFDA), government entities (SNIH, MISA, and MoH), the SCTE, healthcare institutions, academia, multinational pharmaceutical companies, CROs, and clinical investigators.

As participation was based on conference attendance, no predefined selection criteria were applied. However, the meetings brought together diverse stakeholders with relevant expertise across clinical research, healthcare delivery, regulation, and policy. Discussions were facilitated to encourage balanced input from stakeholder groups.

#### Conference proceedings and development of recommendations

Expert input was derived from two national conferences. During the first conference (December 2022), baseline quantitative findings from Phase 1 were presented in plenary sessions. Structured expert panel discussions and facilitated workshops explored key barriers and critical gaps limiting the growth of iCTs. Facilitators ensured balanced participation across stakeholder groups.

The second conference (March 2024) built on these discussions and focused on refining potential solutions. Targeted discussions addressed key strategic themes, including regulatory harmonization, infrastructure development, talent development, patient engagement, and financial incentives.

#### Synthesis of recommendations

Insights from both conferences were retrospectively reviewed and synthesized by the study authors using documented conference materials. Recommendations were developed through iterative review and integration of qualitative insights from conference discussions with quantitative findings from Phase 1 (LongTaal analysis), ensuring alignment with national policy goals (Vision 2030).

Consensus was not established through formal voting or predefined quantitative thresholds. Instead, recommendations reflect areas of broad agreement and recurring themes identified across stakeholder discussions.

#### Considerations on bias

As this qualitative approach relied on conference-derived input, we couldn’t employ formal bias control measures. However, the conferences included diverse stakeholder groups and facilitated discussions to promote balanced contributions. In addition, our recommendations were informed by both multi-sector input and quantitative evidence from Phase I; a mixed approach to avoid relying on a single perspective, given the limited published literature addressing clinical trial infrastructure and strategic planning for iCTs in Saudi Arabia.

### Ethical considerations

Ethical approval was not required for this study, as the research involved the analysis of publicly available aggregate data and the synthesis of expert opinions from public conferences.

## Main findings

This study integrates findings from a mixed-methods approach comprising two complementary phases: a quantitative benchmarking of the Saudi Arabian clinical trial landscape (Phase 1) and a qualitative assessment of expert input derived from conference-based discussions (Phase 2).

### Phase 1: Quantitative landscape analysis

#### Global market share and growth trends

The quantitative analysis covering the 2016–2023 period revealed that while biopharmaceutical companies have globalized clinical research, Saudi Arabia’s participation remains disproportionate to its economic and pharmaceutical standing. In 2023, the Kingdom’s share of globally active iCTs was approximately 0.6% across all phases. Specifically, Saudi Arabia participated in 1.5% of globally active Phase 3 iCTs, which, along with Phase 2 trials, accounted for nearly 95% of active iCT sites in the country (Table 1).

**Table 1:** Comparative analysis of global and Saudi Arabia iCT market share by study phase (2023)

2023 data	Active Studies	% studies	Share of global studies in each phase	Active Sites	% sites	Share of global sites in each phase
KSA Phase 1 and 1/2	1	1%	0.02%	1	0%	0.002%
Global Phase 1 and 1/2	6166	29%		50,945	10%	
KSA Phase 2 and 2/3	12	10%	0.33%	17	6%	0.016%
Global Phase 2 and 2/3	3620	17%		103,603	20%	
KSA Phase 3	51	41%	1.50%	139	46%	0.049%
Global Phase 3	3404	16%		281,627	53%	
KSA Phase 4	4	3%	0.79%	9	3%	0.077%
Global Phase 4	509	2%		11,692	2%	
KSA Phase NA	56	45%	0.74%	136	45%	0.173%
Global Phase NA	7578	36%		78,665	15%	
**KSA all phases**	**124**	**100%**	**0.58%**	**302**	**100%**	**0.057%**
**Global all phases**	**21,277**	**100%**		**526,532**		

iCT, Innovative industry-sponsored clinical trials; KSA, Kingdom of Saudi Arabia; NA, not available

**Fig. 2:**
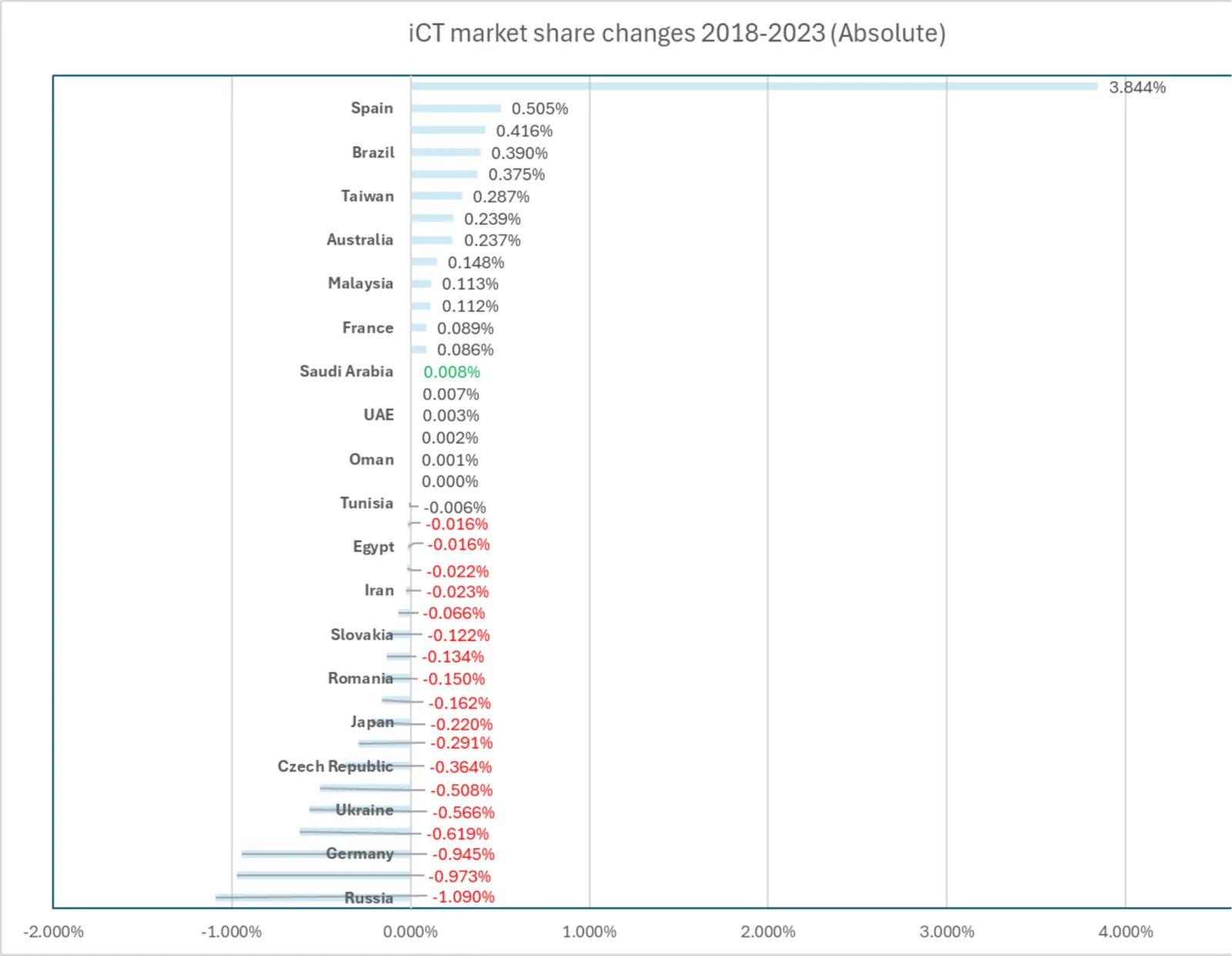

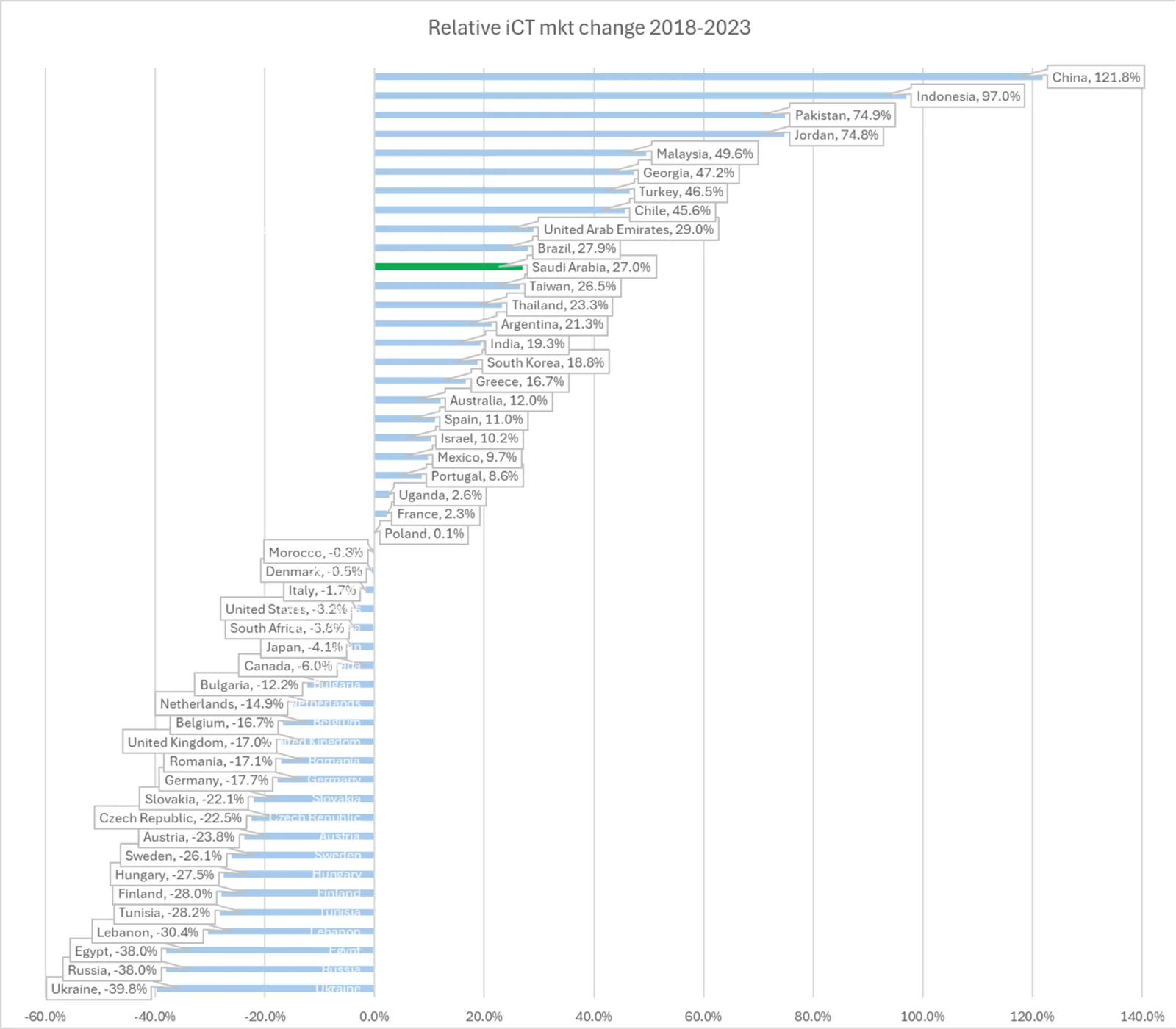
iCT market share trending and relative market growth 2018–2023 for selected countries. The market share was calculated as share 2023–share 2018. Market share in countries was calculated as % of all global active phase 2 and phase 3 iCT sites in the country. The relative growth was calculated as (share 2023/share 2018)—1. iCT: Innovative industry-sponsored clinical trials

Despite the low baseline, the analysis identified a positive growth trajectory. Between 2018 and 2023, the Saudi iCT market expanded by 27% in relative terms, marking five consecutive years of growth and positioning the Kingdom as the 17th globally in relative market growth. This growth trend outperformed several regional peers, although countries like Jordan and the UAE also registered high growth rates from smaller baselines (Fig. 2).

#### Patient representation and research equity

**Fig. 3:**
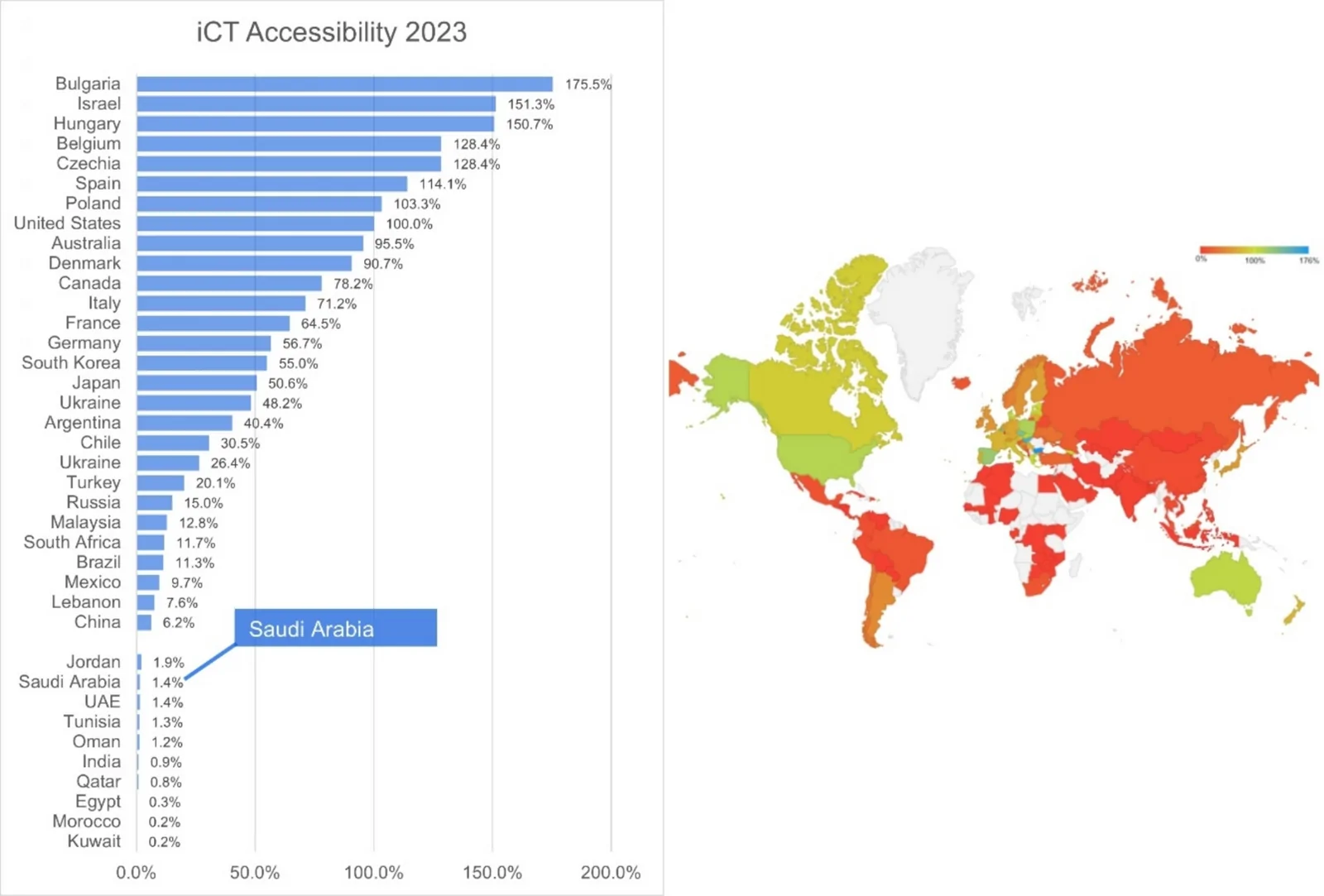
Accessibility to iCTs is calculated as the number of clinical trial sites per 1 million in the population relative to the US levels (US = 100%), (2023 data). iCT, Innovative industry-sponsored clinical trials; UAE, United Arab Emirates

A critical finding presented to the consensus panels was the disparity between pharmaceutical consumption and participation in research. The analysis of Patient Accessibility revealed that Saudi Arabia’s accessibility metric (iCT sites per million population) is approximately 1% of US levels, significantly lower than established markets in North America and Europe (Fig. 3).

**Fig. 4:**
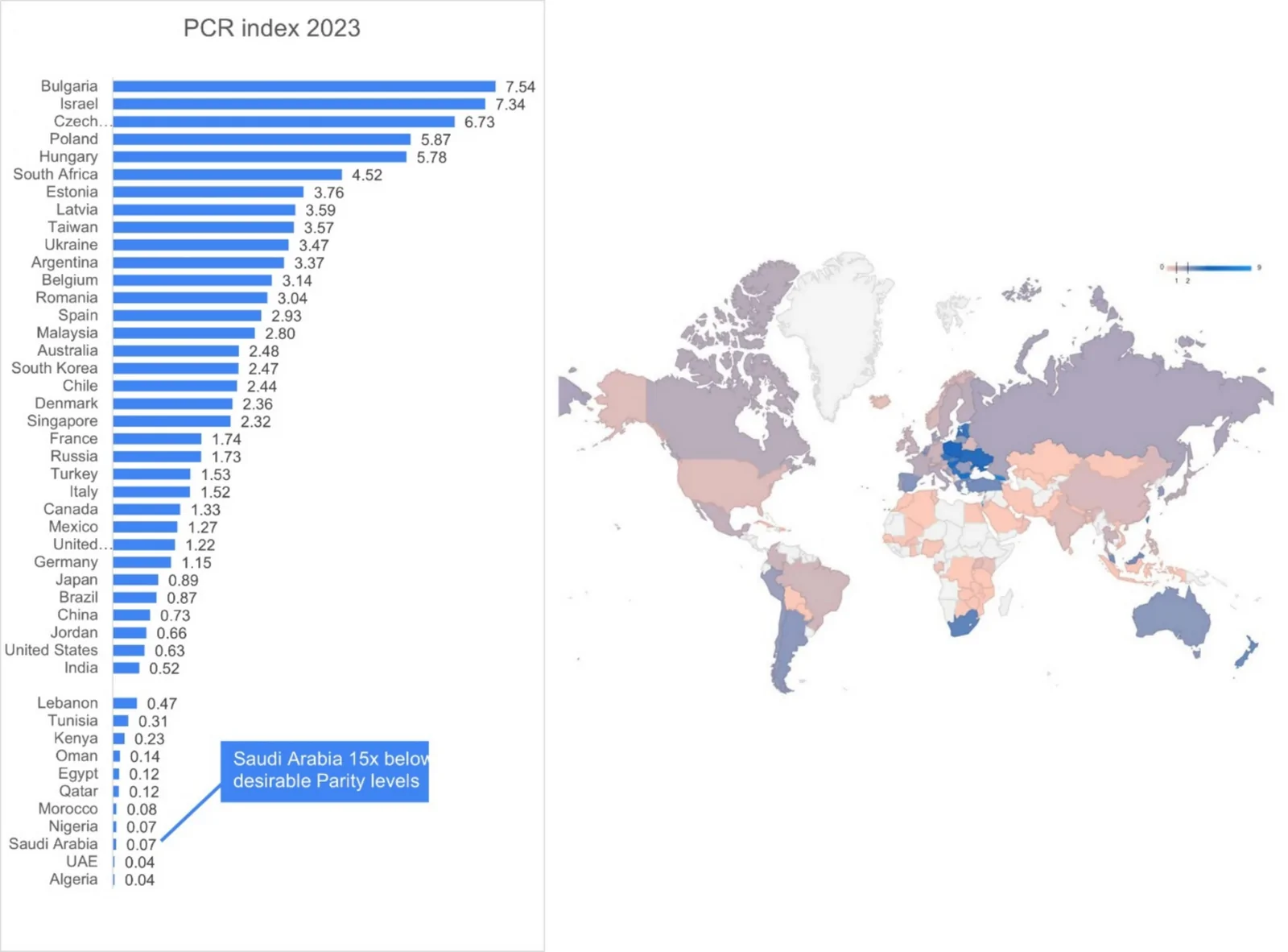
Participation to consumption ratio (PCR) is shown as a global heatmap as well as country ranking of select countries, including Saudi Arabia for definitions and methodology, see Methods (2023 data). UAE, United Arab Emirates

To quantify this gap, the Participation-to-Consumption Ratio (PCR) was calculated. While Saudi Arabia accounted for 0.9% of the global consumption of innovative prescription drugs in 2023, its participation in global iCTs was only 0.06%. This resulted in a PCR of 0.06 or lower, placing Saudi Arabia 92nd among 120 evaluated countries. This 16-fold disparity highlights a significant underrepresentation of Saudi patients in the development of novel pharmaceuticals, raising concerns regarding the availability of safety and efficacy data specific to the local ethnic and genomic profile (Fig. 4).

#### Therapeutic area distribution

The landscape analysis highlighted a divergence between global trends and local activity. While oncology dominates the global iCT landscape, rare diseases accounted for the largest share of trials in Saudi Arabia, followed by cardiovascular and metabolic disorders (Supplementary Table 1). This distribution reflects the high prevalence of specific inherited conditions in the Kingdom. Still, it indicates an underrepresentation in trials addressing other major national disease burdens, such as neoplasms and neurological disorders.

#### Socioeconomic impact assessment

**Fig. 5:**
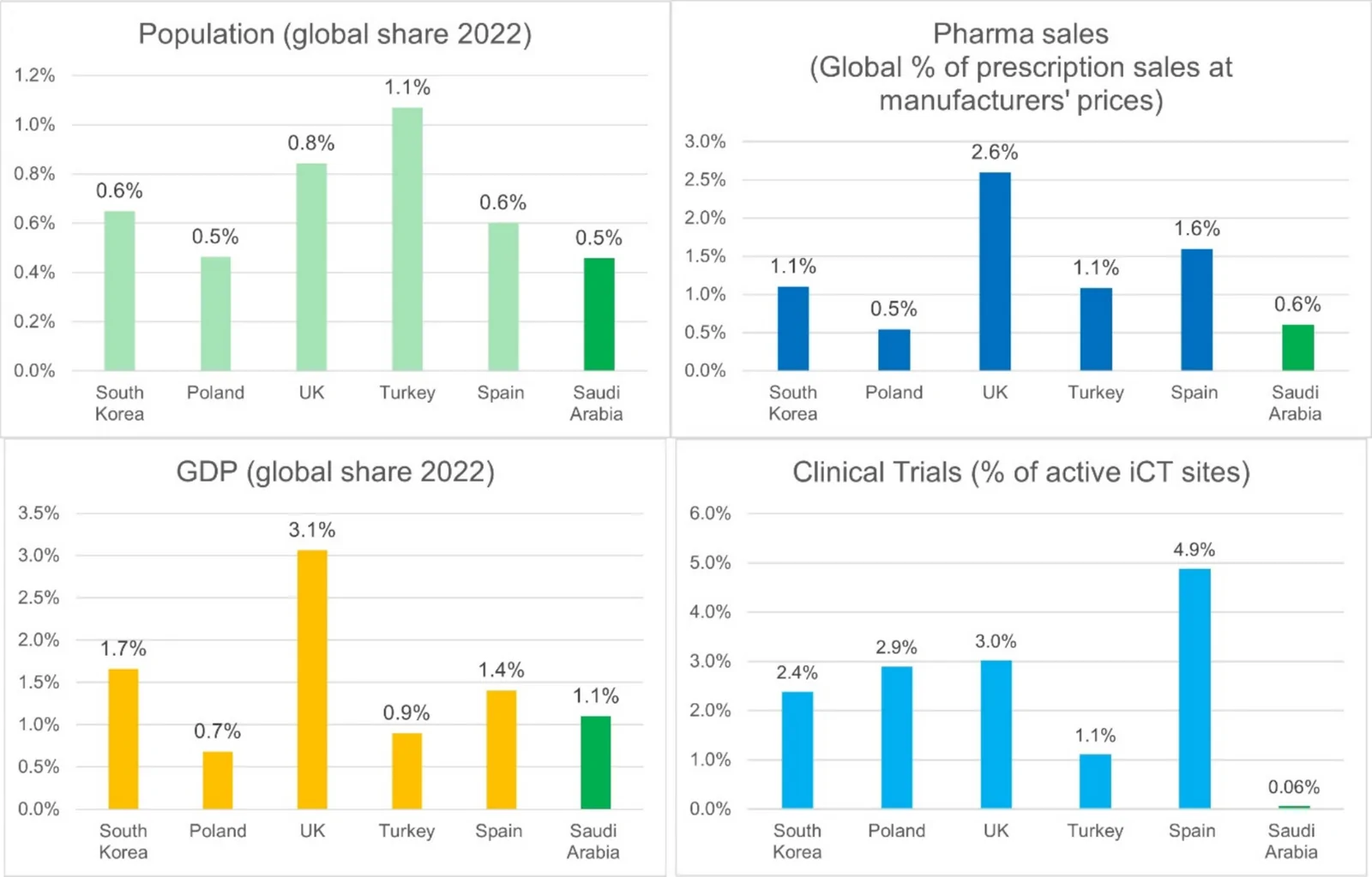
Benchmarking KSA to some other comparable global economies (GDP, Population, Pharma sales, and iCT participation, 2022 data). iCT, Innovative industry-sponsored clinical trials; GDP, gross domestic product; KSA, Kingdom of Saudi Arabia; UK, United Kingdom

**Fig. 6:**
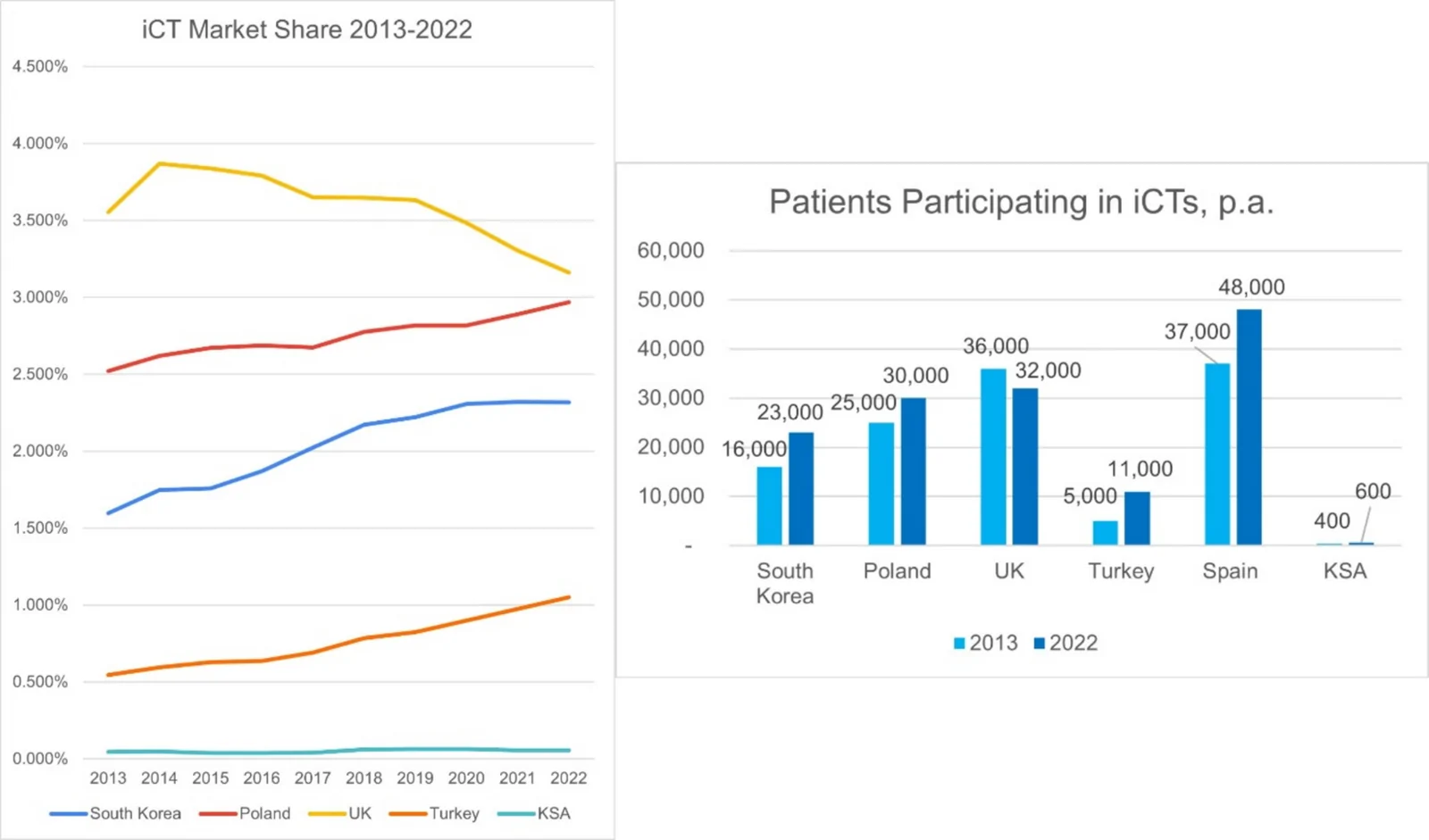
Benchmarking KSA regarding iCT market share and patient participation in iCTs vs other comparable global economies. iCT, Innovative industry-sponsored clinical trials; KSA, Kingdom of Saudi Arabia; UK, United Kingdom

Economic modelling demonstrated that in 2023, iCT activity in Saudi Arabia generated an estimated USD 46 million in R&D investment and supported approximately 200 high-end research jobs. However, comparative benchmarking against economies of comparable size (e.g., South Korea, Poland, Türkiye) indicates that the Kingdom is capturing only a fraction of its potential (Figs. 5 and 6). The models project that achieving parity between consumption and trial participation could increase annual R&D investment to over USD 740 million and create more than 3,000 specialised jobs.

#### Sponsor landscape and activity

A comparative assessment of global iCT sponsors' activity shows that sponsors allocated nearly 1.8% of their international studies and 0.09% of their globally active sites to Saudi Arabia. These figures are notably lower than the allocation to other markets with similar population sizes (2.9%). Although most of the top ten global pharmaceutical companies have some level of activity in Saudi Arabia, several sponsors within the global top 20 and top 30 have no R&D study presence at all (Supplementary Table 2).

Regarding the landscape of non-industry trials, our analysis indicates that 62–72% of Saudi Arabia's active clinical trials in 2020–2023 were led by non-industry (academic) sponsors. While previous studies using ClinicalTrials.gov suggested an even higher academic dominance (80%), analysis of the Saudi Clinical Trials Registry (SCTR) indicates that 63% of applications were industry-sponsored. This discrepancy is likely due to the under-reporting of generic drug trials in global public databases compared to the local SCTR registry. Importantly, despite the high volume of academic trials, the overall site footprint of iCTs generally exceeds that of academic trials, with over half of all active sites in 2022–2023 devoted to innovative iCTs.

#### Growth scenarios and parity goals

**Fig. 7:**
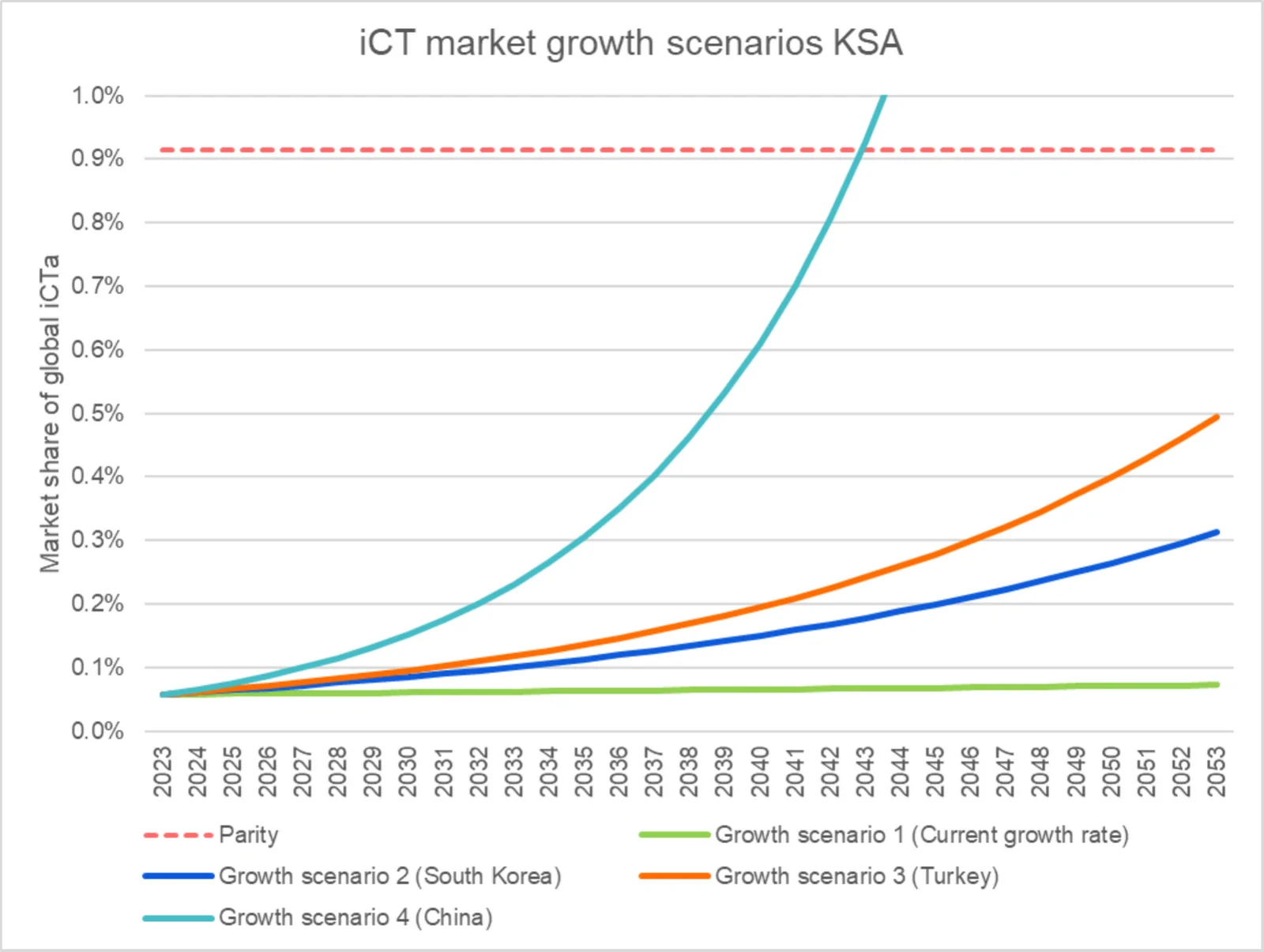
Market growth scenarios for iCTs in KSA. The green line represents the extrapolation of its growth rates in KSA achieved during 2008–2023. Other growth scenarios have been modelled by applying annual growth rates achieved during 2008–2023 in South Korea (blue line), Türkiye (red line), and China (orange line). iCT, *Innovative* industry-sponsored clinical trials; KSA, Kingdom of Saudi Arabia

To assess the timelines required to achieve equitable representation ("parity"), defined as an iCT market share equal to the Kingdom's global pharmaceutical consumption share (> 0.9%), various growth trajectories were modelled (Fig. 7).

If the Kingdom maintains its historical average annual growth rate (2008–2023), parity will not be achieved within this century. Alternative scenarios based on benchmarking markets suggest that matching South Korea's growth rate (5.8%) would achieve parity by 2072, while matching China's rapid expansion (14.9%) would target parity by 2042. To reach parity within 20 years, Saudi Arabia must consistently maintain a 15% annual growth rate. These scenarios underscored to the consensus panels that organic growth is insufficient and that industry sponsors must be incentivized to reallocate a portion of their global pipeline to Saudi Arabia.

### Phase 2: Strategic barriers and priorities

During the discussions at the 1 st and 2nd Regional Clinical Trial Conferences, expert panels reviewed Phase 1 quantitative findings and identified key barriers to iCT growth. These discussions highlighted the following structural challenges:

- Regulatory and Operational Timelines: The panels identified that, despite recent improvements, study start-up timelines (currently 6–8 months) remain a competitive disadvantage compared to high-performance regions achieving 6-month benchmarks.
- Fragmentation of Support: Discussions indicated that the lack of a centralized "one-stop shop" for sponsors creates logistical friction. Experts agreed that a unified body is required to address feasibility, regulatory support, and coordination, thereby reducing administrative burdens for global sponsors.
- Infrastructure and Talent Gaps: While Centres of Excellence exist, discussions suggested that they are not yet sufficiently distributed or standardised. The panels prioritized the need for a larger cadre of trained investigators and study coordinators to support multi-centre trials.
- Incentivization: Stakeholders agreed that current financial incentives are insufficient to drive the necessary behavioural shift among global sponsors. Discussions highlighted the potential need to expand incentives beyond product innovation to include direct operational support for R&D, such as personnel cost reimbursement.

These findings provided the foundation for the strategic roadmap detailed in the subsequent discussion.

The accessibility metric, while indicative, alone is not a true measure of underrepresentation in the development of novel pharmaceuticals. That is why we used PCR to assess and quantify the adequacy of representation of countries' populations in the development of new pharmaceutical products relative to the consumption of commercially available pharmaceutical products (see Methods for definitions and algorithm; Fig. 4).

Saudi Arabia's PCR is below 0.06, placing it 92nd among 120 evaluated countries and corresponding to only roughly 5% of the "desirable" level of participation. This metric underscores a critical shortfall in the inclusion of Saudi patients in drug development efforts, given the Kingdom's relatively high consumption of innovative prescription medicines.

Overall, the underrepresentation of Saudi patients in global clinical trials emphasises the urgency of implementing targeted, country-specific interventions. Authorities, industry stakeholders, and healthcare institutions can explore options such as integrating sub-studies encompassing representative ethnic or cultural cohorts into market authorisation filings and mandating the collection of real-world data post-launch to assess local efficacy and safety. By closing these gaps, the Kingdom would enhance health equity for its patient populations and foster medical innovations tailored to its epidemiological needs, building greater public confidence in new therapies. Robust participation in iCTs is vital for ensuring appropriate safety and efficacy profiles for Saudi patients and realising the broader societal and economic benefits of a thriving clinical research ecosystem.

### Quantifying the socioeconomic impact of iCTs

The analysis reveals that pharmaceutical companies invested an estimated USD 46 million in R&D within Saudi Arabia's iCT ecosystem in 2023. This activity generated approximately 200 high-end R&D jobs and provided over 600 patients access to innovative experimental therapies. While these figures may initially appear encouraging, Saudi Arabia lags in several important measures of clinical trial activity relative to comparable-sized economies (Fig. 5 and 6). As a result, the Kingdom may miss out on potential returns, such as greater R&D investment, more specialised research positions, and broader patient access to innovative treatments. Data suggest that the flow of capital, job creation, and advanced patient therapeutic options could be expanded by as much as 10- to 30-fold.

However, it is worth noting that relying on organic growth in the Saudi iCT market to meet these benchmarks without deliberate and coordinated action may not be realistic. Achieving a more substantial market participation depends on joint efforts and commitments of stakeholders, including governmental bodies, academic institutions, biopharmaceutical companies, CROs, healthcare providers, and patient advocacy groups. The lack of such coordinated initiatives risks failing to realise the full economic and societal advantages of a thriving clinical research environment.

## Recommendations and call for action

Findings derived from the Regional Clinical Trial Conferences provide a unified roadmap for bridging the disparity in Saudi Arabia’s iCT market share. Discussions among expert stakeholders highlighted that achieving the "parity" goal requires a shift from fragmented initiatives to a coordinated national strategy. Based on these insights and the quantitative findings from Phase 1, the authors synthesized a set of strategic recommendations representing the key priorities identified through multi-stakeholder discussions (Table 2).

**Table 2:** Summary of strategic recommendations for expanding iCTs in Saudi Arabia

Strategic Domain	Recommendation	Key Actions and Implementation Mechanisms	Target Outcome	Primary Stake-holder
Governance Framework	Operationalize a centralized national body to act as a "Single Point of Contact" for global sponsors	• Designate a national body to develop an ecosystem among stakeholders and standardize the processes and legislations of CTs in the country • Facilitate feasibility process using aggregated, de-identified EMR data • Oversee study coordination and track milestones	Streamlined feasibility, contracting, and site selection processes for global sponsors	SNIH
Regulatory Competitiveness	Harmonize study start-up timelines to achieve a consistent 6-month benchmark	• Unify CTA processing workflows • Implement centralized IRB approval at the National level • Implement SFDA policies that actively incentivize sponsors to include Saudi patients in pivotal (Phase 2 and 3) studies	Globally competitive timelines aligned with high-performance iCT markets	SNIH/Sites
Infrastructure and Workforce	Establish a distributed network of "Clinical Trial Centres of Excellence."	• Standardize research infrastructure across the MoH, the military, and private hospitals • Implement site-specific referral systems based on patient pathways • Scale training programs for investigators and study coordinators • Qualify research sites and equip them with the essential requirements to conduct high quality clinical trials	Increased capacity to conduct high-quality multicentre studies across the Kingdom	SNIH
Technology Integration	Deploy unified digital platforms to modernize trial conduct	• Adopt AI-driven analytics for recruitment forecasting • Implement interoperable CTMS and telemedicine for decentralized trials	Enhanced data quality, recruitment accuracy, and operational efficiency	Industry Sponsors/ Sites
Financial Incentives	Shift incentives to support the operational footprint of R&D	• Develop business cases for the Ministry of Investment/Finance to reimburse a portion of R&D personnel costs • Move beyond rewarding only product innovation to supporting trial execution	Increased direct foreign investment and sustainability of research sites	SNIH/Sites
Global Engagement	Enhance the international visibility of Saudi Arabia's research ecosystem	• Promote capabilities at major global scientific forums • Leverage academic partnerships to place Saudi experts on Global Steering Committees	Improved reputation and integration into global drug development pipelines	SNIH

*AI* Artificial Intelligence; *CT* Clinical Trial; *CTA* Clinical Trial Agreement; *CTMS* Clinical Trial Management System; *EMR* Electronic Medical Records; *iCT* Innovative industry-sponsored clinical trial; *IRB* Institutional Review Board; *R&D* Research and Development; *SFDA* Saudi Food and Drug Authority; *SNIH* The Saudi National Institutes of Health

### Strategic recommendations

A primary recommendation is the operationalization of a centralized national body to serve as a comprehensive "single point of contact" for global sponsors, effectively functioning as a national Site Management Organization (SMO).

**Fig. 8:**
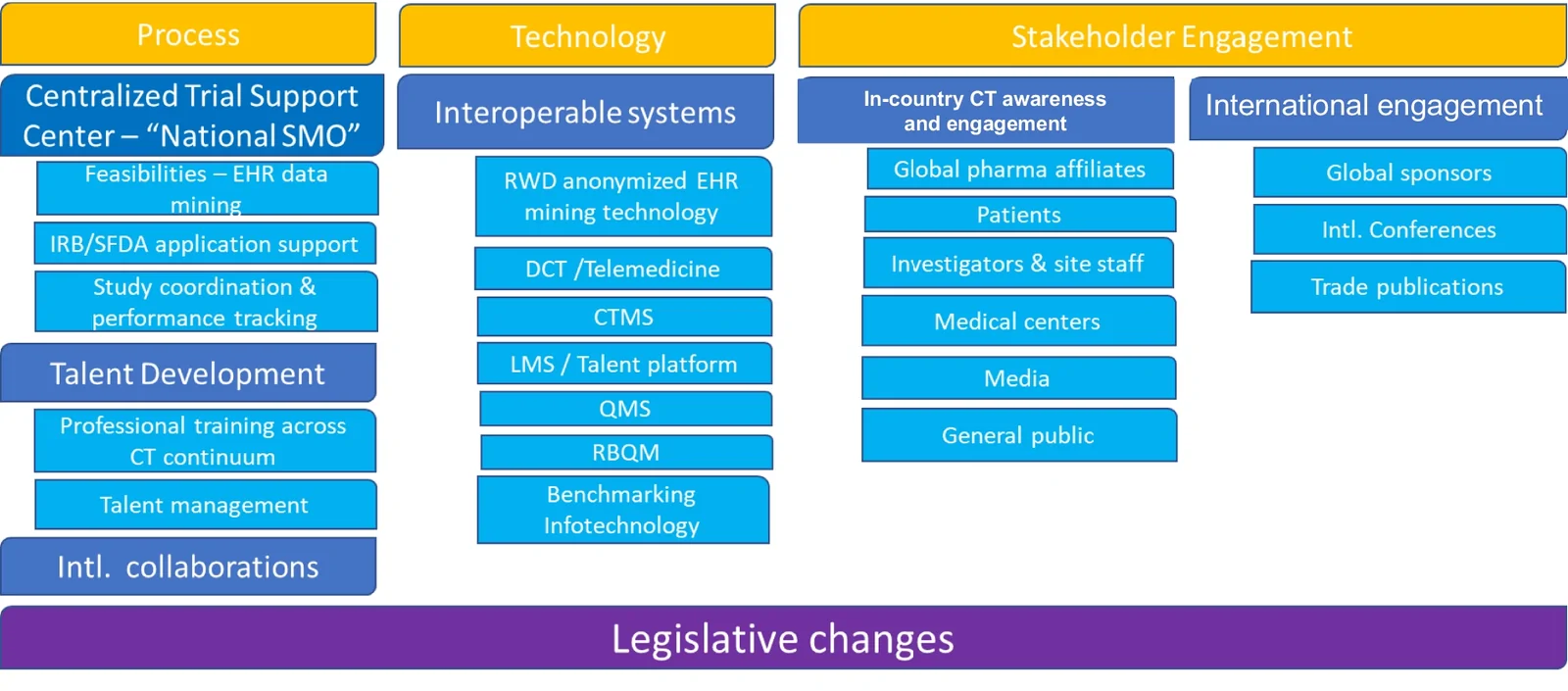
Prescription for iCT Success in KSA: The recommended set of actions, initiatives and process improvements aimed at significantly increasing the share of Global R&D clinical trials in KSA. CT, Clinical Trial; CTMS, Clinical Trial Management System; DCT, Decentralised Clinical Trial; EMR, Electronic Medical Records; IRB, Institutional Review Board; LMS, Learning Management System; QMS, Quality Management System; RBQM, Risk-Based Quality Management; RWD, Real-World Data; SFDA, Saudi Food and Drug Authority; SMO, Site Management Organisation

The framework presented in Fig. 8 illustrates the core functions of this centralized body as informed by the stakeholders discussions and synthesized by the authors:

- Pre-Trial Efficiency: The body should manage feasibility assessments using aggregated, de-identified Electronic Medical Record (EMR) data mining to provide global sponsors with accurate, data-driven recruitment forecasts rather than estimates.
- Regulatory Support: It should facilitate a streamlined submission process to the SFDA and institutional ethics committees, ensuring predictable and competitive timelines.
- Operational Oversight: During the trial phase, the agency would manage study coordination, tracking progress against defined milestones and providing trained study coordinators to sites where resources are scarce.
- Technology Integration: Discussions emphasized the importance of integrating cutting-edge technologies, specifically AI-based data analysis, telemedicine platforms for decentralized trials, CTMS, and Learning Management Systems (LMS), to modernize trial conduct and reduce administrative burdens. While the timeline of implementation remains uncertain, the authors supported an adaptive modernization strategy in which Saudi Arabia could selectively accelerate the adoption of AI-enabled and decentralized clinical trial capabilities alongside strengthening conventional trial infrastructure. This approach was considered a potential competitive advantage for attracting industry sponsors through improved patient recruitment, expanded geographic reach, enhanced operational efficiency, and shorter trial timelines, while maintaining regulatory rigor, data quality, and participant safety.

### Priority focus areas

Beyond the centralised governance structure, the experts discussions prioritized three key operational pillars required to transform the Kingdom into a competitive global hub:

1. Regulatory Competitiveness: Stakeholders identified study start-up timelines as the single most critical differentiator. While progress has been made, further reducing the current 6–8-month study start-up timelines to achieve consistency within 6 months (the benchmark in high-performance iCT markets) is critical. The recommendation is to centralize institutional review board and unify CTA processing and to have the SFDA create policies that actively incentivize sponsors to include local patients in pivotal global studies (Phases 2 and 3). These measures are expected to reduce administrative delays during study start-up, accelerate site activation, and improve the efficiency of patient recruitment for global sponsors.
2. Infrastructure and Workforce Expansion: To support the modelled growth scenarios, discussions highlighted the need for a distributed network of Clinical Trial Centres of Excellence. The proposed roadmap includes:
  Standardizing infrastructure across the MoH, military, and private hospitals to create a "network effect," allowing sponsors to access multiple sites through a single channel.
  Implementing site-specific referral systems based on mapping patient pathways and accessing de-identified EMR data to ensure recruitment targets are met.
  Rapidly scaling the workforce by training investigators and study coordinators to enable high-quality multicentre studies.
3. Strategic Promotion and Incentives: Once foundational improvements are in place, we recommend a proactive international engagement strategy. This includes promoting Saudi Arabia’s capabilities at global scientific forums and leveraging academic partnerships to increase the number of Saudi experts serving on the Steering Committees of Global Multicentre Clinical Trials.

Furthermore, a key recommendation is that the government must look beyond rewarding product innovation. The authors recommend presenting a clear business case to the MISA and the Ministry of Finance to explore mechanisms for reimbursing a portion of R&D personnel costs, thereby incentivizing the operational footprint of clinical research within the Kingdom.

By implementing these measures, Saudi Arabia can serve as a model for other emerging markets seeking to bridge the 'drug development divide,' demonstrating how centralized governance and targeted incentives can rapidly elevate a national research ecosystem.

## Conclusion

Saudi Arabia's Vision 2030 aims to diversify the national economy by reducing its reliance on oil and promoting growth across strategic non-oil sectors, including healthcare, research and development (R&D), and biotechnology. Within this broader agenda, innovative industry-sponsored clinical trials (iCTs) are positioned as a foundational pillar for improving healthcare quality and driving biomedical innovation.

This manuscript integrates a quantitative landscape analysis with expert-informed insights from the 1st and 2nd Regional Clinical Trial Conferences to provide a unified strategic roadmap for the Kingdom. The evidence presented revealed a critical disparity: while Saudi Arabia accounted for 0.9% of global consumption of innovative prescription drugs in 2023, its participation in global iCTs was only 0.06%. This 16-fold gap highlights a significant underrepresentation of Saudi patients in the development of novel pharmaceuticals, given the country's market size.

Addressing this inequity is a public health imperative. Our findings highlight that immediate, strategic action is required to ensure the safety and efficacy of new drugs are evaluated across populations with unique ethnic, genomic, and cultural profiles. Furthermore, closing this gap presents a major economic opportunity: achieving parity between trial participation and pharmaceutical consumption could increase annual R&D investment from ~ USD 46 million to over USD 740 million, creating more than 3,000 high-skilled jobs and expanding patient access to cutting-edge therapies from 600 to over 9,000 annually.

To realize this vision, we endorsed a centralized governance model, recommending that an independent national governing body serve as the single point of contact and one-stop shop for all sponsored CTs. This mandate should encompass pre-trial support, regulatory coordination, technology integration, and advocacy for financial incentives. While the Kingdom's clinical trial ecosystem grew by 27% between 2018 and 2023, reaching 51.4% in the first half of 2025 (SNIH, 2025), more decisive, coordinated measures are required. By implementing the our recommendations outlined in this paper, Saudi Arabia is well-positioned to emerge as a global leader in clinical research, fuelling economic growth and significantly improving healthcare outcomes for its population, thereby directly contributing to the Quality-of-Life Program goals of Vision 2030.

## Supplementary Information

Below is the link to the electronic supplementary material.

Supplementary file1 (DOCX 29 KB)

## Data Availability

All data generated or analysed during this study are included in this published article.
