# Supplementary material for: Bridging the clinical trial gap in Saudi Arabia: a multi-stakeholder conference-based consensus on strategic initiatives for global competitiveness and patient inclusion
Source: Saudi Pharm J. 2026 Jul 21;34(4):44. doi: 10.1007/s44446-026-00099-7 (PMC13388625; doi:10.1007/s44446-026-00099-7)
Supplement: Supplementary file 1 — Supplementary file1 (DOCX 29 KB) [file 44446_2026_99_MOESM1_ESM.docx]

**Bridging the Clinical Trial Gap: Saudi Arabia’s Initiatives to Enhance Global Competitiveness and Patient Inclusion in Industry Clinical Trials**

Majed Al Jeraisy, Pharm.D^1^*, Eva Turgonyi, M.D.^2^, Vladimir Misik, Ph.D.^3,4^, Ahmed Alaskar, MD, FRCPC, FACP^5,6^

^1^Chairman Clinical Trial Management, King Abdullah International Medical Research Center (KAIMRC), Ministry of National Guard-Health Affairs (MNGHA), Riyadh, Saudi Arabia.

^2^Medical Director, GCC (Gulf Countries Cluster) & Pakistan, AstraZeneca, Dubai, United Arab Emirates.

^3^Managing Partner, LongTaal Clinical Research Institute, Bratislava, Slovakia.

^4^Managing Partner DUNA.Logic – Clinical Research Informatics, Hainburg/ Donau, Austria.

^5^Executive Director, King Abdullah International Medical Research Center (KAIMRC), Riyadh, Saudi Arabia.

^6^Consultant & Professor, Adult Hematology & HSCT, King Saud Bin Abdulaziz University for Health Sciences, Ministry of National Guard, Health Affairs, Riyadh, Saudi Arabia.

***Corresponding author**:

Majed Al Jeraisy, Pharm.D

Chairman, Clinical Trial Management, King Abdullah International Medical Research Center (KAIMRC), Ministry of National Guard-Health Affairs (MNGHA), Riyadh, Saudi Arabia.

[Jeraisyma@gmail.com](mailto:Jeraisyma@gmail.com)

**Supplementary Table 1: Comparison of the largest iCTs in Saudi Arabia and globally, 2023**

| **Saudi Arabia** | | | **Global** | | |
| --- | --- | --- | --- | --- | --- |
| Condition (MESH) | Active Studies | Active Sites | Condition (MESH) | Active Studies | Active Sites |
| Haemophilia A | 8 | 15 | Carcinoma, Non-Small-Cell Lung | 701 | 29828 |
| Diabetes Mellitus, Type 2 | 6 | 29 | Breast Neoplasms | 495 | 18132 |
| Crohn Disease | 5 | 16 | Solid Tumours | 366 | 5430 |
| Haemophilia B | 5 | 10 | Advanced Solid Tumour | 334 | 2973 |
| Sickle Cell Trait | 5 | 6 | COVID-19 | 319 | 4231 |
| Asthma | 5 | 23 | Lymphoma | 306 | 8113 |
| Multiple Sclerosis | 4 | 7 | Melanoma | 297 | 8337 |
| Atrial Fibrillation | 4 | 6 | Multiple Myeloma | 288 | 9963 |
| Breast Neoplasms | 4 | 14 | Colorectal Neoplasms | 283 | 6935 |
| Vaso-occlusive Pain Episode in Sickle Cell Disease | 3 | 3 | Diabetes Mellitus, Type 2 | 270 | 8142 |
| Bradycardia | 3 | 5 | Neoplasms | 254 | 6187 |
| Vaso-occlusive Crisis | 3 | 3 | Carcinoma | 217 | 5106 |
| Multiple Myeloma | 3 | 3 | Carcinoma, Squamous Cell | 217 | 5957 |
| Muscular Atrophy, Spinal | 3 | 7 | Small Cell Lung Carcinoma | 217 | 6915 |
| Chronic Myelogenous Leukaemia | 3 | 3 | Heart Failure | 214 | 7339 |
| Carcinoma, Hepatocellular | 3 | 7 | Leukaemia, Myeloid, Acute | 203 | 5911 |
| Mitral Valve Regurgitation | 2 | 4 | Ovarian Neoplasms | 196 | 5043 |
| Obesity | 2 | 5 | Carcinoma, Hepatocellular | 187 | 5869 |
| Tachycardia | 2 | 3 | Adenocarcinoma | 185 | 5607 |
| Leukaemia, Prolymphocytic, B-Cell | 2 | 7 | Advanced Solid Tumours | 181 | 2238 |
| Triple Negative Breast Neoplasms | 2 | 10 | Gastrointestinal Neoplasms | 180 | 3982 |
| Carcinoma, Non-Small-Cell Lung | 2 | 6 | Kidney Diseases | 172 | 5736 |
| Dermatitis, Atopic | 2 | 2 | Prostatic Neoplasms | 172 | 7180 |
| Fabry Disease | 2 | 3 | Alzheimer Disease | 171 | 5443 |
| Heart Failure | 2 | 6 | Dermatitis | 163 | 6430 |
| Cholesterol Ester Storage Disease | 1 | 2 | Osteoarthritis | 160 | 1523 |
| Solid Tumours | 1 | 1 | Arthritis, Rheumatoid | 160 | 4749 |
| Non-Transfusion-dependent Alpha-Thalassemia | 1 | 3 | Ulcer | 155 | 3223 |
| Lipidoses | 1 | 3 | Asthma | 144 | 6378 |
| Seizures | 1 | 2 | Multiple Sclerosis | 143 | 7415 |

**Supplementary Table 2: Top sponsors iCT activities in Saudi Arabia and globally, 2023**

| **Global rank by # studies** | **KSA rank by # studies** | **Sponsor** | **Active Studies Global** | **Active Sites Global** | **Active Studies KSA** | **Active Sites KSA** | **% Global studies in KSA** | **% Global sites in KSA** | **% Studies in KSA** | **% Sites in KSA** |
| --- | --- | --- | --- | --- | --- | --- | --- | --- | --- | --- |
| 1 | 1 | AstraZeneca | 495 | 38,175 | 15 | 62 | 3.0% | 0.16% | 12.0% | 20.5% |
| 3 | 2 | Pfizer | 305 | 15,984 | 10 | 19 | 3.3% | 0.12% | 8.0% | 6.3% |
| 10 | 3 | Sanofi | 215 | 10,741 | 10 | 22 | 4.7% | 0.20% | 8.0% | 7.3% |
| 2 | 4 | Novartis | 388 | 17,778 | 9 | 17 | 2.3% | 0.10% | 7.2% | 5.6% |
| 13 | 5 | Novo Nordisk AS | 159 | 11,774 | 9 | 31 | 5.7% | 0.26% | 7.2% | 10.2% |
| 19 | 6 | Medtronic | 97 | 3,507 | 7 | 9 | 7.2% | 0.26% | 5.6% | 3.0% |
| 4 | 7 | Hoffmann-La Roche | 293 | 20,985 | 5 | 6 | 1.7% | 0.03% | 4.0% | 2.0% |
| 7 | 8 | Johnson & Johnson | 253 | 20,308 | 4 | 9 | 1.6% | 0.04% | 3.2% | 3.0% |
| 15 | 9 | Bayer | 146 | 6,797 | 4 | 4 | 2.7% | 0.06% | 3.2% | 1.3% |
| 25 | 10 | Abbott Medical Devices | 72 | 2,251 | 4 | 10 | 5.6% | 0.44% | 3.2% | 3.3% |
| 26 | 11 | Celgene | 70 | 6,637 | 4 | 14 | 5.7% | 0.21% | 3.2% | 4.6% |
| 41 | 12 | Biogen | 44 | 1,743 | 4 | 10 | 9.1% | 0.57% | 3.2% | 3.3% |
| 5 | 13 | AbbVie | 263 | 18,716 | 3 | 13 | 1.1% | 0.07% | 2.4% | 4.3% |
| 40 | 14 | Alexion Pharmaceuticals, Inc. | 44 | 2,688 | 3 | 7 | 6.8% | 0.26% | 2.4% | 2.3% |
| > 100 | 15 | Agios Pharmaceuticals, Inc. | 12 | 453 | 3 | 8 | 25.0% | 1.77% | 2.4% | 2.6% |
| 9 | 16 | Bristol-Myers Squibb | 224 | 13,459 | 2 | 3 | 0.9% | 0.02% | 1.6% | 1.0% |
| 14 | 17 | Boehringer Ingelheim | 149 | 5,438 | 2 | 7 | 1.3% | 0.13% | 1.6% | 2.3% |
| 27 | 18 | Boston Scientific Corporation | 69 | 1,981 | 2 | 2 | 2.9% | 0.10% | 1.6% | 0.7% |
| 38 | 19 | Ipsen | 45 | 1,982 | 2 | 3 | 4.4% | 0.15% | 1.6% | 1.0% |
| 6 | 20 | Merck Sharp & Dohme LLC | 261 | 21,932 | 1 | 7 | 0.4% | 0.03% | 0.8% | 2.3% |
| 8 | 21 | Eli Lilly and Company | 226 | 18,389 | 1 | 4 | 0.4% | 0.02% | 0.8% | 1.3% |
| 18 | 22 | Amgen | 109 | 8,865 | 1 | 1 | 0.9% | 0.01% | 0.8% | 0.3% |
| 44 | 23 | Vertex | 38 | 931 | 1 | 1 | 2.6% | 0.11% | 0.8% | 0.3% |
| 66 | 24 | Société des Produits Nestlé (SPN) | 26 | 95 | 1 | 3 | 3.8% | 3.16% | 0.8% | 1.0% |
| 79 | 25 | Acerta Pharma BV | 20 | 1,331 | 1 | 3 | 5.0% | 0.23% | 0.8% | 1.0% |
| > 100 | 26 | Actelion | 7 | 836 | 1 | 2 | 14.3% | 0.24% | 0.8% | 0.7% |
| 11 |  | Takeda | 193 | 6,010 |  |  | 0% | 0% | 0% | 0% |
| 12 |  | GlaxoSmithKline | 181 | 9,909 |  |  | 0% | 0% | 0% | 0% |
| 20 |  | Regeneron Pharmaceuticals | 97 | 3,764 |  |  | 0% | 0% | 0% | 0% |
| 22 |  | Incyte | 88 | 5,014 |  |  | 0% | 0% | 0% | 0% |
| 23 |  | Gilead Sciences | 87 | 5,204 |  |  | 0% | 0% | 0% | 0% |
| 29 |  | Daiichi Sankyo | 64 | 4,441 |  |  | 0% | 0% | 0% | 0% |
| 30 |  | Astellas | 63 | 2,966 |  |  | 0% | 0% | 0% | 0% |
|  |  | **Grand Total** | **6,998.0** | **330,558** | **125** | **303** | **1.8%** | **0.092%** | **100.0%** | **100.0%** |
